# The Use of Targeted Cytokines as Cancer Therapeutics in Glioblastoma

**DOI:** 10.3390/cancers15143739

**Published:** 2023-07-23

**Authors:** Moloud Sooreshjani, Shashwat Tripathi, Corey Dussold, Hinda Najem, John de Groot, Rimas V. Lukas, Amy B. Heimberger

**Affiliations:** 1 Department of Neurological Surgery, Feinberg School of Medicine, Northwestern University, Chicago, IL 60611, USA; 2 Department of Neurology, Feinberg School of Medicine, Northwestern University, Chicago, IL 60611, USA; 3 Department of Neurosurgery, University of California San Francisco, San Francisco, CA 94143, USA; 4 Malnati Brain Tumor Institute of the Robert H. Lurie Comprehensive Cancer Center, Feinberg School of Medicine, Northwestern University, Chicago, IL 60611, USA; 5 Department of Neurosurgery, Northwestern University, Chicago, IL60611, USA; 6 Simpson Querrey Biomedical Research Center, 303 E. Superior Street, 6-516, Chicago, IL 60611, USA

**Keywords:** glioma, cytokines, tumor microenvironment

## Abstract

**Simple Summary:**

Despite multi-modal treatment consisting of surgery, chemotherapy, and radiation, glioblastoma inevitably recurs due to its diffuse infiltrative nature. Anti-tumor immune responses, supported by pro-inflammatory cytokines, that can seek out remote cancer vestiges will likely become part of the therapeutic armamentarium but will require thoughtful selection, combinatorial vetting, and innovative delivery strategies.

**Abstract:**

Cytokines play an important role in regulating the immune response. Although there is great interest in exploiting cytokines for cancer immunotherapy, their clinical potential is limited by their pleiotropic properties and instability. A variety of cancer cell-intrinsic and extrinsic characteristics pose a barrier to effective treatments including cytokines. Recent studies using gene and cell therapy offer new opportunities for targeting cytokines or their receptors, demonstrating that they are actionable targets. Current efforts such as virotherapy, systemic cytokine therapy, and cellular and gene therapy have provided novel strategies that incorporate cytokines as potential therapeutic strategies for glioblastoma. Ongoing research on characterizing the tumor microenvironment will be informative for prioritization and combinatorial strategies of cytokines for future clinical trials. Unique therapeutic opportunities exist at the convergence of cytokines that play a dual role in tumorigenesis and immune modulation. Here, we discuss the underlying strategies in pre- and clinical trials aiming to enhance treatment outcomes in glioblastoma patients.

## 1. Introduction

Glioblastoma isocitrate dehydrogenase wild type (GBM IDHwt) is a highly infiltrative malignancy that is poorly controlled by the standard of care that includes surgery, radiotherapy, chemotherapy, and alternating electrical fields [1,2,3,4]. Objective response rates (ORR) are very low and are influenced, in large part, by the specific mechanism of action of the therapeutics and their effects on imaging parameters more than on direct tumor cytotoxicity [5]. Current treatment approaches for GBM remain challenging due to tumor heterogeneity [6], an immune-suppressive tumor microenvironment (TME) [7], and the highly infiltrative nature of these tumors [8]. Cytokines are soluble small molecules that mediate the interactions between immune and non-immune cells in the TME and either support pro- or anti-inflammatory responses [9]. Targeted delivery of immune modulatory cytokines through either gene- or cell-based strategies [10,11,12,13] may limit adverse effects related to the systemic administration and enhance the efficacy of the treatment [13]. Herein, we focus on cytokine-targeted therapy that mediates crosstalk between cancer and immune cells that have yet to be fully investigated or integrated into treatment strategies. 

Efforts to use immune responses to control cancer date back to 1891, when William Coley attempted treatment of sarcoma patients using mixtures of live and inactivated *Serratia marcescens* and *Streptococcus pyogenes* [14]. More recently, immunotherapy has become a well-integrated component of the standard of care. This success is grounded in an understanding of the specific mechanisms of immune dysregulation in cancer. Work from Allison et al. has been foundational for inducing immune responses to overcome disseminated cancer and provide prolonged duration of responses [15,16]. Multiple immunotherapy strategies have been and continue to be investigated in clinical trials including combinations utilizing immune checkpoint inhibitors [17,18,19,20] and oncolytic viruses [21,22,23,24,25,26]. However, current immunotherapy strategies have limited benefits in GBM [27]. This lack of responsiveness indicates the need to expand the current approaches designed to treat GBM.

## 2. Modulation of Tumor Immunogenicity

**Figure 1:**
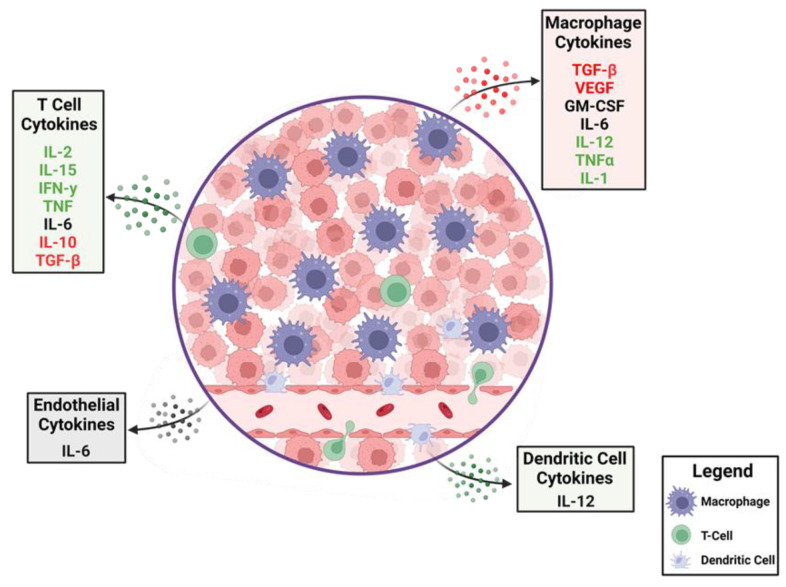
The dynamics of cytokines in the glioblastoma tumor microenvironment (TME). A cartoon depiction of the cytokines that modulate anti-tumor immune responses. Production of immune-suppressive cytokines shown in red are counterbalanced by pro-inflammatory cytokines shown in green. Cytokines that have different immunological roles depending on context are shown in black. A variety of cells within the TME elaborate these cytokines with some, such as macrophages, being abundant, whereas T cells are relatively rare.

**Figure 2:**
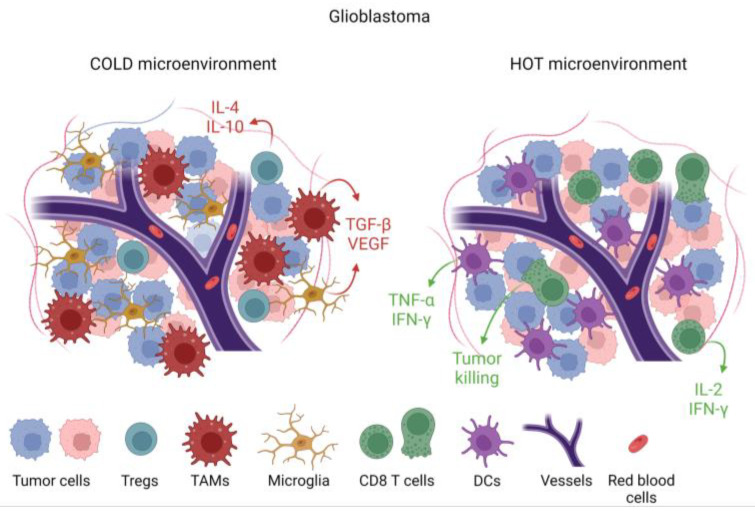
Immunological features of tumors based on cellular and cytokine composition within the tumor microenvironment (TME). Tumors that are devoid of cytotoxic T cells and pro-inflammatory cytokines such as IL-2, IFN-γ, and TNF-α, but with immune-suppressive cytokines such as TGF-β and immune-suppressive cells such as tumor-associated macrophages (TAMs), are designated as immunologically cold. This cold TME is associated with microglia infiltration. In a hot TME, which is rare in glioblastoma, there would be abundant CD8 cytotoxic T cells and dendritic cells alongside pro-inflammatory cytokines.

GBM is a heterogeneous disease that develops a complex TME composed of infiltrating immune cells, vasculature, and fibroblasts exposed to various soluble factors affecting tumor growth [28]. These various factors within the TME determine phenotypic features and treatment outcomes. Cancer cells create an immunosuppressive microenvironment through a variety of mechanisms including inducing immune-suppressive macrophages/microglia [29] and downregulation of antigen presentation [30]. The presence of myeloid-derived suppressor cells (MDSCs) is one of the mechanisms that promote immunosuppressive TME and likely inhibits effective immunotherapy [31]. MDSCs migrate as immature cells from the bone marrow to tumors, where they differentiate into mature macrophages and dendritic cells [32,33]. MDSCs inhibit activation and proliferation of cytotoxic T cells [34] through increased expression of arginase-1 [35], resulting in increased secretion of IL-10 [36] and TGF-β [37]. Tumor-associated microglia/macrophages (TAM) impose additional constraints on anti-tumor immunity [38] by secreting low levels of pro-inflammatory cytokines [39] and compromising T cell function as summarized in Figure 1 and Figure 2 [40]. This immune suppression is further compounded by a paucity of T cells within the TME through sequestration in the bone marrow [41] and irreversible T cell exhaustion [42]. 

## 3. Cytokine Biology

Cytokines are secreted proteins that engage the extracellular domains of cell surface receptors and regulate immune response and homeostasis [9]. Cytokines can be classified based on their roles as pro- or anti-inflammatory cytokines [43] or on cellular origin (Table 1). Type 1 (cellular response) cytokines are secreted by CD4+ Th1 and type 2 (humoral response) cytokines are produced by CD4+ Th2 cells [44]. Although the immune regulatory effects of cytokines make them compelling candidates for cancer immunotherapy, undesirable side effects and short serum half-life can restrict clinical implementation [45]. Cytokine pleiotropy, which refers to the ability of cytokines to act on different cell types in the immune system and peripheral tissues, is also a challenge for clinical translation because of off-target effects [46]. Multiple immunomodulatory cytokines have or are being investigated for clinical use, including TGF-β, CSF-1, IL-2, IL-7, IL-10, IL-12, IL-18, IL-21, IL-22, and IFN-α, some of which include glioma patients (Table 2). Only IFN-α and IL-2 have received U.S. Food and Drug Administration (FDA) approval for cancer treatment [47]. There has been limited experience with high-dose IL-2 in GBM patients after one subject had a fatal outcome secondary to herniation associated with marked T cell tumor infiltration that has not been reported. Human interferon alpha 2b (*IFN*-*α2b*) was approved for the treatment of hairy cell leukemia in 1986 and recombinant IL-2 for treating melanoma and renal cancers in 1992 [47]. With these treatments, severe side effects can include capillary leak syndrome and cytokine release syndrome, leading to death in some patients. In many instances, the concentration of the cytokine leads to different effects including unwanted off-target toxicities. As opposed to conventional chemotherapy in which the highest tolerated doses are typically used, efforts need to be directed at the identification of the appropriate dose for the desired physiological result in the case of cytokines. As such, the management of cytokines, including toxicities, is a more subtle process with titration of the dose in contrast to more standard pharmacologic management of an “on/off switch” approach. As such, the management of cytokines is a different concept when juxtaposed with cytotoxic chemotherapy where the intention is to maximize cytotoxicity. Given the toxicity of cytokine-based therapies, considerable effort has been focused on targeting cytokines through cytokine-producing viral vector gene therapy and adoptive transfer of cytokine-producing cells. Below, we discuss specific promising cytokine-based approaches undergoing investigation in GBM. We first discuss the targeting of pro-tumoral cytokines followed by a discussion of approaches using pro-inflammatory anti-tumoral cytokines.

## 4. Targeting Pro-Tumoral Cytokines

### 4.1. Targeting Transforming Growth Factor β (TGF-β)

TGF-β is a cytokine with pleiotropic effects which may play an important role in anti-tumor immune responses [77]. TGF-β supports stem-like self-renewal and suppression of immune response [78]. TGF-β expression, presumably in the context of the above-described effects, is associated with glioma development and progression [79]. In turn, targeting this cytokine is a rational therapeutic approach. A non-randomized phase 1/2 clinical trial (NCT01220271) showed the safety and tolerability of LY2157299, a small molecule inhibitor of TGF-β receptor type I, in combination with temozolomide and radiation in newly diagnosed high-grade gliomas [80]. However, treatment of patients with LY2157299 and lomustine did not improve the overall survival (OS) relative to monotherapeutic lomustine in patients with recurrent GBM [81]. Another approach for targeting TGF- β involves the use of bintrafusp alfa, a bifunctional protein consisting of an antibody blocking PD-L1 and TGF-β trap [82]. Because PD-L1 can be expressed on some types of cancer cells which prevents T cells from killing, targeting two distinct mechanisms of tumor-mediated immune suppression may show an additive or synergistic effect. Partial responses were observed in a phase 1 trial of this agent in conjunction with radiation and temozolomide in patients with recurrent GBM [82]. Because PD-L1 is not frequently expressed on GBM [83,84], this strategy likely needs to be considered in the context of selected patients. In addition, the size of the therapeutic molecule requires consideration with respect to its ability to adequately cross the blood–brain barrier (BBB) at adequate concentrations to treat the tumor. Antisense nucleotides are another means for targeting TGF-β. These (AP12009) have been investigated in a non-randomized phase 2 trial in which they were directly administered into recurrent tumors using convection-enhanced delivery (CED). Partial and complete responses were observed [85]. There are a number of technical challenges currently associated with CED [86] which limit scalability and dampen the enthusiasm for later-stage clinical investigations. 

### 4.2. CSF-1

Colony-stimulating factor-1 (CSF-1) is a glycoprotein cytokine that functions through the receptor CSF1R [87] and regulates the differentiation of myeloid progenitors into dendritic cells, monocytes, and macrophages [88]. One of the most frequent immune cells within the TME are TAMs. The cells can become polarized to the M1 and M2 states [89,90] in which the M1 state exerts a pro-inflammatory, anti-tumor response [91] and the M2 state promotes tumor growth, invasion, metastasis, and resistance to therapy [92]. TAM-directed therapies using CSF-1 and CSF1R inhibitors have been tested in preclinical models of gliomas [93,94], as well as in clinical studies. A phase 2 trial (NCT01349036) of pexidartinib (PLX3397), a CSF1R inhibitor, in recurrent GBM was well tolerated but did not improve progression-free survival (PFS) [95]. Similarly, a combination of pexidartinib, radiation therapy, and temozolomide did not improve median PFS or OS in newly diagnosed GBM [96]. This lack of effect may be due to, at least in part, compensatory mechanisms such as CSF2-driven macrophage resistance or phosphatidylinositol 3-kinase [97]. 

### 4.3. The Paradoxical Targeting of the Granulocyte-Macrophage Colony-Stimulating Factor (GM-CSF) for Glioblastoma

GM-CSF is a hemopoietic growth factor and is responsible for the expansion and activation of macrophages and granulocytes [98]. GM-CSF modulates cell maturation proliferation and survival. GM-CSF boosts immune responses by promoting T and B cell expansion and differentiation and dendritic cell maturation, proliferation, and migration. It is from this immunological perspective that GM-CSF has been used in oncology clinical trials including a wide variety of peptide vaccine strategies for GBM patients. Notably, GM-CSF is elevated in cancer patients [99]. In glioblastoma, GM-CSF and its receptor can promote tumor progression likely through upregulating anti-apoptotic and pro-angiogenic signals via the activation of the signal transducer and activator of transcription 3 (STAT3) signaling pathway or by increasing the expression of VEGF and its receptor [100,101]. In the tumor environment, tumor cells, and tumor-associated microglial cells secrete GM-CSF [102,103,104]. Inhibiting GM-CSF thereby can suppress cancer cell growth and metastasis [103]. GM-CSF has been used in multiple large vaccine trials for GBM which could have had both beneficial and detrimental effects [1,72]. Given the dual pro-cancer and pro-inflammatory roles of GM-CSF, monotherapy inhibitors will likely not be tested in the context of glioma. 

## 5. Utilizing Anti-Tumoral Cytokines

### 5.1. Virus-Based Cytokine Expression

Virotherapy is an evolving class of immunotherapies based on the selective replication of these viruses in cancer cells to trigger tumor antigen presentation, immune activation, and subsequent tumor cytotoxicity [20,21,22]. Initiation and activation of apoptosis in the cancer cells and the induction of type I IFN is the underlying mechanism of these types of viruses. Viruses can also be devised to elaborate a variety of cytokines to modulate the immune system that thereby mediates the anti-tumor effect. The first oncolytic virus approved by the FDA in 2015 for the treatment of metastatic melanoma was talimogene laherparepvec (T-VEC), an engineered herpes simplex virus-1 that expresses human GM-CSF [105,106]. A series of preclinical studies have shown that cytokine-armed viruses can enhance immune response and provide additional survival benefits in glioma-bearing mice. For example, a virus expressing IL-4 prolonged survival in tumor-bearing mice [107] and one expressing a single-chain variable fragment of the epidermal growth factor receptor (EGFR) antibody conjugated to CCL5 increased the infiltration of innate and adaptive immune cells [108].

A number of cytokine-elaborating viruses have been tested in GBM [21,22,23,24] but tumor heterogeneity and the immune-suppressive TME have likely compromised clinical effectiveness thus far. Ad–RTS–hIL-12 is an adenoviral vector expressing IL-12 controlled by binding of an orally administered ligand, veledimex [62]. Safety, tolerability, and feasibility were demonstrated in a phase 1 monotherapy trial in recurrent high-grade glioma. The ability to measure extra-CNS spill-over of IL-12 and its downstream product IFN-γ was demonstrated via elevated serum concentrations. Based on preclinical studies, the intracranial concentration of cytokines was likely substantially higher than what could be measured in the serum. Post-treatment resected tumor tissue demonstrated an increase in T cell infiltration of the tumor. This approach has been further investigated in conjunction with PD-1 blockade in the phase 1 [61] and phase 2 settings [109]. As discussed earlier, the highest level of IL-12 production did not appear to be the optimal dose for impacting survival and in turn was not utilized as the phase 2 dose. Two other IL-12-based viral vector gene therapy approaches are currently under investigation in gliomas. Ad-TD-nsIL12, a human adenovirus with three genes deleted and expressing human non-secretory IL-12, was developed to minimize IL-12 toxic effects [110]. A phase I Ad-TD-nsIL12 trial (NCT05717699, NCT05717712) in pediatric patients with diffuse intrinsic pontine glioma is currently recruiting patients in China. Another phase 1/2 trial (NSC 733972) is now enrolling patients with high-grade gliomas to study the combination of M032, a genetically engineered HSV-1 expressing IL-12, with pembrolizumab.

### 5.2. The Addition of IFN-α with the Standard of Care Temozolomide

IFN-α can inhibit tumor cell proliferation, enhance the cytotoxic activity of macrophages and natural killer (NK) cells, and prevent the formation of blood vessels in tumors [111]. A multi-center randomized phase 3 clinical trial enrolled 199 patients with high-grade gliomas. After receiving standard radiation therapy with concurrent temozolomide, patients were randomized to receive either temozolomide or temozolomide with IFN-α. The median OS of patients in the temozolomide plus IFN-α group was 26.7 months, which was longer than that in the standard of care group of 18.8 months (*p* = 0.005). Seizure and influenza-like symptoms were more common in the combination group [48]. The potential benefit was consistent with a prior study that demonstrated that a pegylated formulation had some benefit in addition to temozolomide [112]. However, a prior phase III study of 275 randomized high-grade glioma patients had demonstrated that IFN-α did not improve time to disease progression or OS when added to treatment with radiation therapy and carmustine. Patients treated with IFN-α experienced more fevers, chills, myalgia, somnolence, confusion, and neurological deficits [49]. The differences in outcomes between these trials may have been a function of the combination with the type of chemotherapy. 

### 5.3. Systemic Cytokine Therapy in Conjunction with Brain Tumor Vaccines

The objective of cancer vaccines is to stimulate adaptive immunity against tumor antigens to control tumor growth [113]. The first cancer vaccine approved by the FDA was sipuleucel-T (Provenge), which is a personalized vaccine developed using ex vivo activated peripheral-blood mononuclear cells co-incubated with a recombinant fusion protein (PA2024) to control asymptomatic metastatic castration-resistant prostate cancer [114]. Various types of GBM vaccines have been developed that are usually administered in conjunction with GM-CSF [71,115,116,117,118,119,120,121,122,123]. Thus far, they have not demonstrated an improvement in survival. Newer strategies involve the co-administration of additional cytokines to augment the potential activity of glioma vaccines. For example, IL-12 was shown to improve the therapeutic efficacy in preclinical murine models bearing intracranial gliomas treated with dendritic cells loaded with GL261 mRNA [124]. Several different approaches are being investigated with all appearing safe and having acceptable tolerability thus far.

### 5.4. Cell-Based Therapies

Cell-based therapies rely on genetically modified immune cells such as T, NK, and B cells. Adoptive transfer of genetically engineered chimeric antigen receptor (CAR) T cells demonstrated success in hematologic malignancies and melanoma with six CAR T cell therapies having received FDA approval [125]. While preclinical studies of CAR T therapy were effective in brain tumor control [126,127], overall response rates have been low, likely because of antigen heterogeneity [128,129] and the immune-suppressive TME [130,131]. CAR T therapy may have the ability to reprogram TME and thus may be a compelling partnering approach with other treatment modalities [131]. Improving CAR T therapy can be achieved by engineered expression of cytokines or their receptors to enhance T cell activation, proliferation, and trafficking. In preclinical testing, disialoganglioside (GD2)-targeting CARs engineered with constitutively active IL-7 receptor or IL-15, enhanced survival in GBM xenograft models [132,133]. In another approach, the expression of CXCR1 or CXCR2 in CAR T cells improved trafficking in a GBM model [134]. An upcoming phase 1 trial (NCT05353530) has been designed to assess the safety and feasibility of IL-8 receptor-modified CD70 CAR T treatment in CD70+ and MGMT-unmethylated GBM patients. IL13 receptor alpha 2 (IL13Ra2) is a monomeric receptor of IL-13 [135] that is expressed in ~70% of GBM patients. IL-13Ra2 is associated with higher-grade glioma and poor prognosis [136]. Data from the clinical experience of IL-13Ra2 CAR T intracranial administration supported the safety of CAR T in patients with recurrent GBM [137]. 

NK cells have also been evaluated in the treatment of gliomas [138,139]. NK cells, a key component of innate immunity, facilitate cell lysis by degranulation achieved by the activating receptor NK group 2 member D (NKG2D) [140], killer cell immunoglobulin-like receptor (KIR), and coactivating/adhesion DNAX-activating molecule (DNAM-1) [141]. Because NK cells become deactivated by TGF-β in the immune-suppressive TME of GBM [138], these cells are co-administered with IL-2 and a TGF-βR1 inhibitor (NCT05400122) or are genetically modified so that the TGF-βR is deleted (NCT04991870) in ongoing clinical trials for colorectal adenocarcinoma and GBM patients, respectively. 

### 5.5. Cytokines Associated with Toxicity in GLIOMA Patients

Distinct elevated serum cytokines may be associated with side effects in glioma patients. In one study, plasma profiling of patients treated with the antiangiogenic agent aflibercept in 28 patients with recurrent GBM revealed that changes in IL-13 from baseline to 24 h predicted on-target toxicities. Increases in IL-1β, IL-6, and IL-10 at 24 h were significantly associated with fatigue [142]. 

### 5.6. The Modern Era of Monitoring Intratumoral Cytokines

Under most circumstances, cytokines of CNS tumor patients are measured in the periphery, and these are likely not fully representative of intra-CNS, including intratumoral, concentrations. To determine both the absolute intratumoral concentrations and to follow the longitudinal kinetics, microdialysis catheters can be implanted with minimal risk [143]. This type of analysis is important since it may also identify those subjects that are showing early signs of response, whereas those who do not demonstrate immune effector responses could be spared further ineffective therapy or an alternative therapy based on the changes in the tumor microenvironment. This is contingent on the conditions that cytokines alone would be biologically meaningful as a biomarker of response and that the captured time point for analysis coincides with the therapeutic monitoring period. 

### 5.7. Modulating Cytokines in Glioma Preclinical Model

There are substantial preclinical efforts to use cytokines, especially in adoptive cellular strategies. For example, IL-7 expressed by CAR T improved the survival outcome in a GBM murine model [144]. In another model, IL-15-modified CAR T also improved median survival [127]. Thus far, it is unclear in what specific contexts these cytokine modifications of CAR T cells should be optimally used, the prioritization of which ones, or the combinations. A key limitation is the distribution of adaptive immune therapies through a complex heterogeneous TME. In addition to the delivery of cytokines using viral vectors, an alternative strategy would be the deposition of cells elaborating cytokines and/or chemokines in the TME using BBB opening ultrasound [26]. This type of strategy allows for large molecules to be deposited into the glioblastoma TME. Our group engineered antigen-presenting cells to express CXCL10. These were deposited into the TME of gliomas and markedly increased the number of T cells in the TME and increased median survival [145]. Moving forward, one could engineer off-the-shelf cells that have been transduced with a variety of pro-inflammatory cytokines that are deposited into the TME using BBB opening ultrasound for sustained delivery. 

## 6. Future Directions

Clinical trials investigating the therapeutic effect of cytokines in glioblastoma patients have demonstrated signals of biological response. Cytokine-modulated therapy in GBM will likely evolve as a combinatorial strategy with other immune therapies. The pathophysiology of the cancer and the mechanisms of resistance will prioritize the selection of the cytokines likely in the context of the specific types of immune therapy. Although studies have provided significant insights into the outcomes of GBM patients treated with cytokine modulation, there are significant areas of investigation needed to fully optimize this strategy. One could argue that targeting key hubs such as p-STAT3 that control many immune-suppressive cytokines might be a more rational strategy in GBM. Recent studies have highlighted the importance of IL-33 in GBM progression. Secretion of IL-33 from glioma cells recruits TAM and microglia and promotes a pro-tumorigenic environment [146]. Phospho-proteomic analysis revealed that IL-33+ tumors have a high expression of p-STAT3. STAT3 is a transcriptional regulator of IL-10 [147] and TGF-β [129]. STAT3 inhibits proinflammatory cytokines and dampens the generation of antigen-dependent T cells and T cell proliferation [148]. A BBB penetrant inhibitor of STAT3 is being advanced into phase II studies in combination with radiation [149,150].

Given the multiplicity and various roles of cytokines in the GBM TME, it is unlikely that a single cytokine-focused therapy will result in patient benefits greater than the well-established standard of care. As such, strategically targeting the most immunosuppressive cytokines while pairing an immune checkpoint blockade is the next logical step. To specifically focus on the biology of the TME, particularly the abundance of myeloid lineage cells preventing adaptive immune responses, would enhance the potential for this therapeutic approach to achieve success [151]. As such, pairing established inhibitors of immunosuppressive signaling in myeloid cells with pro-inflammatory checkpoint blockades could be a worthy avenue. Designing brain-penetrant, homing, and myeloid-specific combinatorial approaches is a daunting task and requires the combined collaboration of vastly different areas of scientific expertise. Nanoparticles modified with mannose residues, the binding partner of the canonical immunosuppressive M2 surface receptor CD206, have been shown to target immunosuppressive TAMs while also carrying a payload capable of reversing their immunosuppression [152]. This strategy is proof-of-principle that targeting and reversing immunosuppression in the TME can be cell-specific. To apply this to GBM, we then need to address the spatial challenges of treating the tumor and overcoming the BBB. BBB opening delivery strategies could be a way to deposit cytokine-elaborating cell factories. The contents and cytokines were selected based on specific TME components assayed during biopsy or through circulating biomarkers, to precisely address the TME of the individual tumor and to work in synergy with therapeutic response to existing checkpoint blockades [153]. Novel cytokines that relate to tumor-associated myeloid cells including osteopontin (OPN) [154], and macrophage inhibitory factor (MIF) [155,156], seem to address the above parameters in that they both regulate myeloid cell trafficking and the immunosuppressive phenotypes in the TME of GBM. 

### Key Strategic Decisions for the Scientific Community

- Which cytokines should be prioritized for use and why?
- How can optimal cytokine concentration/dose be established and what is the best strategy for modulation?
- Are there some contexts in which certain cytokines should be used relative to others?
- If we were to devise a cellular biofactory for the deposition of various cytokines into the TME, what should be prioritized?
- Are there some cytokines that should be explored next for GBM that have not been thus far?

## 7. Conclusions

Although various studies have provided valuable insight into cytokine-based therapy, significant efforts need to be directed toward selection of cytokine(s) in various indications, optimization of combinatorial strategies, delivery strategies, and companion biomarkers. 

## Figures and Tables

**Table 1:** Cytokine control of the immune system.

Mediator	Cellular Source	Function
IL-1	Macrophages, epithelial cells	Pro-inflammatory, macrophage, and Th17 cell activation
IL-2	T cells	Effector T cell and regulatory T cell growth factor
IL-4	Th-cells	T and B cell proliferation and B cell differentiation
IL-6	Macrophages, T cells, endothelial cells	Both pro-inflammatory and immune suppressive, increased antibody production
IL-8	Macrophages, epithelial cells	Recruitment of neutrophils
IL-9	Th9 cells	Activation of mast cells
IL-10	Regulatory T cells, Th9 cells	Immune suppressive, inhibition of Th1 cells
IL-11	Fibroblasts, neurons	Immune suppression
IL-12	Dendritic cells, macrophages	Activation of Th1, induction of interferon from cytotoxic T cells and NK cells
IL-15	CD8 T cells, NK cells	Expansion of memory CD8 and NK cells
IL-17	Th17 cells, NK cells	Promotes neutrophilic inflammation
IL-18	Monocytes, macrophages, dendritic cells	Pro-inflammatory, activation of the Th1 pathway
IL-33	Macrophages, dendritic cells, mast cells, epithelial cells	Pro-inflammatory, amplification of Th1 and Th2 cells, activation of NK cells
IFN-γ	Th1 cells, cytotoxic T and NK cells	Pro-inflammatory and activates macrophages
Tumor necrosis factor	Macrophages, T cells, NK cells	Pro-inflammatory increases vascular permeability
GM-CSF	Macrophages, T cells, NK cells, and endothelial cells	Pro-inflammatory but glioma propagating
VEGF	Macrophages	Angiogenesis
TGF-β	Macrophages, T cells	Immune suppressive
CXCL9	Monocytes, endothelial cells	Recruitment of Th1, NK, and dendritic cells
CXCL10	Monocytes, endothelial cells	Recruitment of macrophages, Th1, and NK cells
CXCL12	Mesenchymal stem cells	Chemotactic for T cells
CCL2	Macrophages, dendritic cells	Recruitment of Th2, monocytes, and dendritic cells
CCL3	Monocytes, neutrophils, dendritic cells	Recruitment of macrophages, Th2, NK, and dendritic cells
CCL4	Macrophages, neutrophils, endothelium	Recruitment of macrophages, Th1 cells, NK, and dendritic cells
CXCL13	B cells	Recruitment of B cells, CD4 T, and dendritic cells

**Table 2:** Chemokine clinical trials in glioma patients.

Mediator	Phase	Therapeutic Benefit	Side Effects	Reference
IFN-α	3	Increase in overall survival in combination with the current standard of care	Seizures and flu-like symptoms	[48]
	3	No benefit in combination with radiation and carmustine	Fevers, chills, myalgia, somnolence, confusion, and neurological deficits	[49,50,51]
IFN- α-2a	2	No benefit	Dermatological effects	[52]
IFN-α-2b (PEG-Intron)	2	No benefit in DIPG patients	Well tolerated	[53]
IFN-β	2	No benefit in combination with the current standard of care	Increased neutropenia	[54,55,56,57,58,59]
IFN-γ	2	No benefit	Well tolerated	[60]
IL-12	1	Safety	Well tolerated	[61,62,63]
CXCR4 inhibitor	1	Safety	Well tolerated	[64]
CSF-1 inhibitor	2	No benefit	Well tolerated	[65]
TGF-βR1	2	Safety	Preserved T cell counts	[66,67]
TGF- βR2	2	No benefit	Seizures, edema	[68]
TNF-α	1	Safety	Well tolerated	[69,70]
GM-CSF	3	No benefit	Well tolerated	[1,71,72]
IL-2	1	No benefit	Fatigue, edema	[73,74,75,76]
